# Supplementary material for: scRNA-seq and scATAC-seq reveal that Sertoli cell mediates spermatogenesis disorders through stage-specific communications in non-obstructive azoospermia
Source: eLife. 2025 May 15;13:RP97958. doi: 10.7554/eLife.97958 (PMC12081002; doi:10.7554/eLife.97958)
Supplement: Supplementary file 1. [file elife-97958-supp1.docx]

| **Cluster** | **NOA1** | **NOA2** | **NOA3** | **OA1** | **OA2** |
| --- | --- | --- | --- | --- | --- |
| Germ_cells | 1654 | 792 | 0 | 2329 | 2124 |
| Leydig | 515 | 3892 | 3625 | 572 | 352 |
| Sertoli | 90 | 22 | 123 | 239 | 286 |
| Endothelial | 108 | 223 | 188 | 150 | 224 |
| PMCs | 544 | 226 | 1427 | 701 | 641 |
| Smooth_muscle | 278 | 380 | 325 | 108 | 212 |
| Schwann_cell | 6 | 22 | 37 | 3 | 11 |
| Macrophage | 116 | 153 | 577 | 137 | 67 |
| Mast_cells | 2 | 11 | 17 | 6 | 0 |
| T_cells | 19 | 110 | 153 | 15 | 15 |
| B_cells | 0 | 22 | 4 | 0 | 1 |
| Plasma_cells | 0 | 33 | 2 | 0 | 0 |

**Supplementary Table S1** **The number of different kinds of cells in five samples in scRNA-seq.**
