# Supplementary material for: scRNA-seq and scATAC-seq reveal that Sertoli cell mediates spermatogenesis disorders through stage-specific communications in non-obstructive azoospermia
Source: eLife. 2025 May 15;13:RP97958. doi: 10.7554/eLife.97958 (PMC12081002; doi:10.7554/eLife.97958)
Supplement: Supplementary file 3. [file elife-97958-supp3.docx]

**Supplementary Table S3** **The number of Sertoli cell subtypes in five samples in scRNA-seq.**

| **Sertoli cell subtype** | **NOA1** | **NOA2** | **NOA3** | **OA1** | **OA2** |
| --- | --- | --- | --- | --- | --- |
| Sertoli-1 | 8 | 5 | 17 | 78 | 129 |
| Sertoli-2 | 7 | 5 | 78 | 10 | 20 |
| Sertoli-3 | 14 | 1 | 0 | 42 | 33 |
| Sertoli-4 | 8 | 3 | 0 | 44 | 27 |
| Sertoli-5 | 18 | 0 | 0 | 24 | 30 |
| Sertoli-6 | 28 | 7 | 0 | 17 | 16 |
| Sertoli-7 | 3 | 1 | 28 | 5 | 14 |
| Sertoli-8 | 4 | 0 | 0 | 19 | 17 |
