# Supplementary material for: scRNA-seq and scATAC-seq reveal that Sertoli cell mediates spermatogenesis disorders through stage-specific communications in non-obstructive azoospermia
Source: eLife. 2025 May 15;13:RP97958. doi: 10.7554/eLife.97958 (PMC12081002; doi:10.7554/eLife.97958)
Supplement: Supplementary file 4. [file elife-97958-supp4.docx]

**Supplementary Table S4** **The number of Sertoli cell subtypes in five samples in scATAC-seq.**

| **Sertoli cell subtype** | **NOA1** | **NOA2** | **NOA3** | **OA1** | **OA2** |
| --- | --- | --- | --- | --- | --- |
| Sertoli-1 | 26 | 2 | 27 | 22 | 113 |
| Sertoli-2 | 276 | 21 | 229 | 803 | 449 |
| Sertoli-3 | 155 | 9 | 18 | 182 | 237 |
| Sertoli-4 | 5 | 0 | 0 | 2 | 3 |
| Sertoli-5 | 3 | 0 | 0 | 4 | 7 |
| Sertoli-6 | 3 | 0 | 2 | 5 | 12 |
| Sertoli-7 | 5 | 0 | 3 | 3 | 6 |
| Sertoli-8 | 1 | 0 | 1 | 1 | 0 |
