# Supplementary material for: scRNA-seq and scATAC-seq reveal that Sertoli cell mediates spermatogenesis disorders through stage-specific communications in non-obstructive azoospermia
Source: eLife. 2025 May 15;13:RP97958. doi: 10.7554/eLife.97958 (PMC12081002; doi:10.7554/eLife.97958)
Supplement: Supplementary file 5. [file elife-97958-supp5.docx]

**Supplementary Table S5 Patient characteristics in this study.**

|  | **OA1-P1** | **OA2-P2** | **NOA1-P3** | **NOA2-P4** | **NOA3-P5** | **OA3-P6** | **NOA4-P7** | **NOA5-P8** |
| --- | --- | --- | --- | --- | --- | --- | --- | --- |
| *Demographics* | | | | | | | | |
| Age | 26 | 30 | 32 | 29 | 26 | 43 | 28 | 26 |
| BMI | 20.2 | 25.8 | 24.2 | 28.7 | 25.4 |  |  |  |
| Ob/Gyn History | No/0 | Yes/one son | Yes/0 | Yes/0 | Yes/0 | No | Yes | No |
| Smoking | Quit smoking for 3 months | 10 years | No | No | Quit smoking for 6 months | No | No | No |
| Alcohol use | Yes | Yes | Yes | No | No | No | No | No |
| *Hormone profile* | | | | | | | | |
| FSH (mIU/mL) | 2.98 | 2.2 | 20.96 | 30.86 | 9.21 | 4.06 | 19.3 | 22.16 |
| LH (mIU/mL) | 6.86 | 4.3 | 7.64 | 8.72 | 5.13 | 6.95 | 8.9 | 10.75 |
| PRL (mIU) | 291 | 329 | - | 235.7 | 257.7 | 12.1 | 462 | 34.59 |
| T (ng/mL) | 5.3 | 13.57 | 3.46 | 4.18 | 1.53 | 18.2 | 3.3 | 4.29 |
| E2 (pg/mL) | 29.35 | 125.2 | 31 | 30.67 | 31.7 | 39 | 38 | 29.21 |
| *Volume of testis (mL)* | | | | | | | | |
| Left | 15.1 | 13.3 | 6.9 | 3.9 | 5.43 | 13.11 | 5.85 | 4.6 |
| Right | 14.2 | 16.1 | 5.3 | 5.8 | 6.47 | 13.96 | 5.41 | 5.5 |
| *Spermatic cord* | | | | | | | | |
| Left | 1.9 mm; regurgitation; 3 s | Non-regurgitation | 1.5 mm; Non-regurgitation | 2.5 mm; regurgitation; 5 s | 1.5 mm; Non-regurgitation | Normal | Normal | Normal |
| Right | 1.5 mm; Non-regurgitation | Non-regurgitation | 1.5 mm; Non-regurgitation | Non-regurgitation | 1.5 mm; Non-regurgitation | Normal | Normal | Normal |
| *Other* | | | | | | | | |
| Ejaculatory duct | Widening (inner diameter approx. 2mm) | Normal | Normal | Normal | Normal | Normal | Normal | Length 10mm, thickened duct wall |
| AZF | Normal | Normal | Normal | Normal | Normal | Normal | c deletion | Normal |
